# Endeavouring interplay: a grounded theory study of how nurse educators’ work with simulation-based learning

**DOI:** 10.1186/s12912-023-01546-9

**Published:** 2023-10-11

**Authors:** Kari Røykenes, Monika Kvernenes, Tove Giske

**Affiliations:** 1 Institute of Nursing, Faculty of Health Studies, VID Specialized University, Bergen, Norway; 2 Center for Medical Education and Department of Clinical Medicine, Faculty of Medicine, University of Bergen, Bergen, Norway

**Keywords:** Nurse educator, Facilitator role, Simulation-based learning, Grounded theory

## Abstract

**Background:**

Simulation-based learning is frequently used as a teaching and learning strategy in nursing and other health professions educations, and educators have a key role as facilitators. This facilitator role provides them with a particularly relevant perspective to help us understand and theorize around the essence of simulation-based learning, and how it is approached. This study aims to explore nurse educators’ experiences and strategies in simulation-based learning.

**Method:**

Data were collected in 2018-2021 using in-depth interviews with eight nurse educators. Transcripts were analysed through constant comparison using Classical Grounded Theory approach.

**Result:**

The participants' main concern was how to *Maximize students' learning-space in simulation*. To resolve this, four strategies were identified: *legitimizing simulation, self-development, preparing students,* and *tailoring simulation*. Legitimisation, and self-development were found to be important prerequisites for developing the learning space and were therefore defined as the background or context of the theory. Nurse students were the focus of the two remaining categories, preparing students and tailoring simulation, and are thus defined as being in the foreground of the theory. The dynamics of these four strategies were captured in the Grounded theory of *Endeavouring interplay*.

**Conclusion:**

The theory of *Endeavouring interplay* illustrates the complexity educators are encountering when aiming to optimize simulation as a learning space for nurse students. The strategies used are adapted to the organisational climate, available resources and context, and include striving to legitimize simulation, pursue self-development in the role as facilitator, help students prepare for simulation-based learning, and tailor the simulation to both contextual factors and individual student needs.

**Supplementary Information:**

The online version contains supplementary material available at 10.1186/s12912-023-01546-9.

## Introduction 

Simulation-based learning (SBL) is frequently used as a teaching and learning strategy in nursing and other health professions educations [1]. SBL allows students to rehearse realistic scenarios in a risk-free learning environment, scaffolded by a trained facilitator and procedural rules, including a reflective debriefing session. The purpose is to provide students with participatory learning experiences that are intended to prepare them for clinical practice. Although popular with students and teachers, the method remains critiqued for lacking a theoretical foundation and for having a weak evidence base for its strengths as a pedagogical approach [2–4].

Educators have a key role as facilitators in SBL training. This role provides them with a particularly relevant perspective to help us understand and theorize around the essence of SBL, and how it is approached. This study aims to explore nurse educators’ experiences and strategies in facilitating SBL.

## Background

SBL is resource intensive; consequently, many nursing programs have interest in gathering evidence to support the effectiveness and added value of this teaching method. There is ample research on SBL focusing on student learning outcomes [5, 6], students’ satisfaction [7], how to optimize the learning environment, including the significance of psychological safety and debriefing [6, 8, 9], facilitators’ characteristics [10, 11] and implementation of SBL in nursing curricula [12]. Also, there are many reports on how SBL is used within various subfields of nursing [13–15]. Furthermore, SBL has proven effective in helping students’ integration of skills and theorical knowledge [1]. The challenge is that SBL is resource demanding (staff, time, rooms, and equipment) and thus hard to find space for in the tight schedules of theoretical and practical education in universities [16].

There is, however, a shortage of studies reporting on how facilitators experience SBL. A study from 2011 found that nurse facilitators valued simulation because of its ability to provide students with standardized learning experiences and that simulation ensured consistency across the curriculum [17]. They also appreciated that “*scenarios could match course content and enhance learning of the material*” and that the “*scenarios could be used to allow students the opportunity to intervene in high-risk but low-occurrence situations in the acute care setting*” ([17], p8). Paige and Morin [18], found that nurse facilitators were concerned with facilitating a process of discovery for the students during simulation. Similar findings are reported by Krogh, Bearman and Nestel [19] who report that facilitators wove values, fundamental beliefs, and creativity into their debriefing practices. Other key aspects of SBL that have been highlighted are the importance of tailoring scenarios to students’ needs, attention to creating a safe environment, and the promotion of reflection [20].

Research reports on challenges associated with the educators’ role as facilitators, which may influence the nurse educators' self-confidence and practice. Structural barriers, including finding time for planning, funding for adequate rooms and equipment, and human resources, have been identified in several studies [16, 17, 21]. Furthermore, the complex nature of facilitating SBL leaves facilitators with the task of meeting high expectations communicated both by students [22] and in guidelines for best practice [23]. A study exploring nurse educators’ perceptions of using simulation to teach fundamental care accentuates this as they describe facilitating as both a “privilege but also a challenge” since they felt obligated to act as role models and convey fundamental care as key to nursing [24].

Finally, the literature reports various pedagogical dilemmas associated with facilitating SBL. This includes attending to group dynamics, assigning role characters and pairing of students [17, 18], and how to allow for mistakes without inducing feelings of defeat, which is seen as detrimental for the students’ learning experience [18]. A recent study also reports how facilitators use complex strategies to accommodate students’ emotions as part the simulation learning experience [25].

These characteristics of SBL are not exclusive to nurse education. Similar findings are reported in interprofessional studies amongst nurses and physicians [26].

With this background, our study aimed at exploring nurse educators’ experiences and strategies in facilitating SBL. The overall purpose was to develop a theoretical model for understanding the complex nature of SBL.

## Method

### Design

We chose the inductive methodology of Classical Grounded Theory (CGT) as our research strategy [27]. CGT is considered a suitable approach when the research purpose is to uncover the meanings of people’s social actions, interactions, and experiences. In CGT, the researchers enter the field of study with a deliberately open attitude to explore the substantive area, which in our study was educators facilitating SBL in nursing education. Interviews typically start with a grand open question so that the participants can talk freely about their experiences and how they understand their role. The researchers seek to discern what the main concern is for the participants [27]. Understanding the main concern is important because it captures the driving forces of what the participants do within the area of study. Data collection and analyses are done concurrently. In a process of constant comparative analysis of new data, the researchers ask the question: “What is this a study of?” [27], and thus code for strategies and conditions in the data. Codes are built into broader categories, and the analyses seek one overarching code that covers all the variation in data, which is called the core category. The core category explains what the participants do to solve their main concern. As the participants’ main concern and core category are developed consecutively throughout the analysing process, the interview questions become more focused and detailed, allowing the theory to become dense and saturated [28].

### Context

In Norway, where this study was conducted, the 3-year bachelor program in nursing must adhere both to Norwegian and EU legislation, because nurses graduating in Norway have access to the EU job market. According to the EU directive, at least half of the nursing program must be clinical studies with direct patient contact. SBL is widely used, and although there is broad consensus that SBL cannot replace clinical practice, it helps prepare students for clinical studies.

### Participants

The sample was purposive, guided by the consecutive analyses. We aimed to achieve variation in experience amongst the participants. Eight nurse educators with 1 to 10 years of experience as SBL facilitators participated, and the majority were described by colleagues as experts in the field. Seven of them were female. Participants were between 30–60 years old. They were recruited from four universities where the majority had large cohorts of students (200+ per year), and they worked in five different campuses in Norway. They all had professional backgrounds as nurses and had diverse clinical experience before they started working in higher education. All eight were trained as facilitators, and one had studied SBL at master’s level.

### Ethical considerations

The data collection was approved by the Norwegian centre for research data (NSD) (reference number: 155276). All participants were initially invited by the first author via e-mail with an invitation to take part in the study. After receiving a positive response, prospective participants were presented with written information about the project. Before the interviews were conducted, participants signed a written consent stating that they were informed about the purpose of the study and that their partaking was voluntary. It was also highlighted that they could withdraw from the research at any time and that the data would be treated confidentially.

### Data collection and analysis

Data were collected from individual interviews. The interviews started with an invitation to share their experiences: *Please tell us about your experiences with using simulation*. Examples of follow-up questions are: *Can you tell us about when you started using simulation-based learning?* and *What makes you still use this method?* Five of the interviews were performed in person at a place chosen by the participants at their university. Due to the Covid situation and long geographic distances, three interviews were conducted as video interviews using the Teams software. As the analysis of interviews progressed, the authors discussed suitable new candidates for interviewing, what areas to explore in more depth (theoretical sampling), and what new questions needed to be asked. Examples of added questions were: *Can you give examples of sequences of simulation that worked well and why you think they did? How do you work to advocate and legitimize SBL at your university?* The interviews lasted between 45–60 min and were recorded and consecutively transcribed verbatim.

After the first interview was transcribed, the three authors analysed it independently and wrote memos, before we met to discuss initial codes and tentative main concerns. We discussed what strategies and conditions could be identified. Our initial analyses of the first interviews were taken into account when we planned for the next interviews. After the third interview, we were able to formulate a tentative main concern and a core category. The next three interviews were subjected to open coding as we continued to analyse the transcripts asking ourselves questions such as:* What are the participants talking about? How do they understand and handle simulation? What challenges do they describe and how are they resolved? What conditions do they talk about, and which strategies could be identified in the data?*

After the fifth interview, the researchers came together for a 2-days’ workshop where we, through constant comparison of our data, gradually saturated and built up the concepts of the evolving theory. Based on this analysis we moved on to theoretical sampling and selective coding from the sixth interview onwards.

In the final two interviews, we continued with initial open questions, followed by probing in areas where we needed more data. Hence, we could move forward in developing the indicators of the concepts in the emergent theory. Since the eighth interview only gave nuances to previous findings but no new perspectives, we discontinued further data collection. We agreed on the main concern of our participants and the core category. We tried out different models and ways to weave our findings together and discussed how we could best present the substantive theory of “Endeavouring interplay.”

Classical Grounded Theory (CGT) is an inductive method where the theory is based on a constant comparative approach between the data, the analysis, and the theory. As the theory develops, new questions arise based on previous data collection and analysis. Theory generating using CGT is a method that takes time to develop. Data in present study were therefore collected over a 4-years period, between October 2018 and October 2021.

## Results

Participants’ main concern was to *maximize students’ learning space* in simulation.

Before we present our finding, we will shortly summarize three conditions that affect how nurse educators maximize students’ learning space.

### The nursing student

Students’ former experiences in using simulation as a learning method and their emotional states when entering simulation are significant conditions for educators in facilitating SBL. These conditions impact how the educators plan, tailor, and conduct SBL to maximize students’ learning.

### The organisation

The organisation in which SBL takes place is an important premise supplier that affects educators’ opportunities to carry out and implement SBL. Significant conditions include how simulation is embedded in the curriculum and what support and resources are made available by program leaders and colleagues. As SBL is resource demanding to do well, educators dedicated to SBL stay continuously alert so that simulation is not reduced or removed from nursing education.

### The educator

The third condition affecting the maximalization of students’ learning space is the educators themselves. Their experiences as nurses, teachers, and facilitators affect how they approach simulation.

#### Nurse educators’ understanding of SBL

In this study, simulation was seen as a powerful way for students to learn. Facilitators argued that it provides hands on training and allows students to learn in environments that closely resemble actual nursing practice. Participants highlighted that SBL contains four elements: First, students acquire introductory knowledge and train basic skills and procedures before coming to simulation. Second, it is crucial that teachers agree on the learning outcomes on which they can develop the scenarios and thus prepare the students for SBL. The third element is the simulation situation itself. Finally, the debrief, where students and the facilitator reflect on the actual event and the learning that can be drawn from it, was considered a significant part of SBL. The informants also highlight the differences between role-play and SBL, as simulation is understood to be more systematic since it follows a defined templet.

Participants argued that SBL is a method that can be used for diverse scenarios and not just limited to acute situations. One said it was just the imagination that sets limits for simulation, and examples of scenarios suggested were related to communication, death and dying, and interaction with next of kin.

#### Endeavouring interplay

The core category in this study is named Endeavouring interplay, and through the four main categories, and their attached sub-categories, it explains how nursing educators work hard and sometimes struggle to maximize students’ learning space (see Table 1). These four main categories are: legitimizing simulation, self-development, preparing students, and tailoring simulation. Legitimisation, and self-development are found to be important prerequisites for developing the learning space and are therefore defined as the background or context of the theory of Endeavouring interplay. Nurse students are the focus of the two remaining categories, preparing students and tailoring simulation, and are thus defined as being in the foreground of the theory.

**Table 1:** Overview of the core category with main and sub-categories, and their positioning in back- and foreground of the theory

Core category	Main categories	Subcategories
Endeavouring Interplay	**Background**	
	Legitimizing simulation	i) Lobbying for curriculum changes ii) Advocating simulation iii) Strategically choosing collaborators iv) Fighting for resources v) Holding leaders accountable
	Self-development	i) Asking for feedback from peers ii) Pursuing formal education iii) Participating in courses and conferences iv) Connecting with the SBL community v) Participating in research
	**Foreground**	
	Preparing students	i) Informing students about learning objectives ii) Agreeing on rules for simulation iii) Helping students familiarize with equipment iv) Highlighting the non-evaluative nature of simulation
	Tailoring simulation	i) Establishing fidelity and authentic environments ii) Attending to students’ emotions iii) Encouraging student activity iv) Promoting reflection in debrief v) Highlighting decisive learning episodes vi) Facilitating re-simulation

##### Legitimizing simulation

There are barriers that thwart the implementation and maintenance of simulation in nurse education. Resistance from colleges toward implementing the method, together with limited resources, are significant barriers. To overcome these barriers, educators use different strategies. Lobbying for curriculum changes is done at different educational levels. One strategy is to get engaged in the institution’s regular curriculum evaluations and work for simulation implementation in curriculum revisions. In this process, the nurse educators make efforts to clarify where in the curriculum SBL is suitable as a learning method. Committing colleagues to the idea of simulation by highlighting that SBL is not just a general learning method one can choose if one likes and discussing the need to reduce established learning methods to allow time for simulation is important. As one participant expressed: *“If my colleagues say: “There is no room for more in this subject” I usually reply: “If you are to use a new learning method you must take away something else”*. Another strategy when lobbying for curriculum changes is to use simulation in externally financed projects and then use project outcomes to leverage the implementation of SBL in the local curriculum.

When advocating simulation, various strategies are used to convince colleges and leaders who are sceptical, critical, or indecisive about simulation as a learning method. One approach is to demonstrate simulation outcomes, by inviting colleagues who are not familiar with the method to observe or participate in SBL sessions. By demonstrating students’ outcomes in simulation, educators illustrate that simulation can be seen as the “missing link” between theory and practice. Another strategy used to advocate simulation is to encourage colleagues to participate in facilitator courses or other formal simulation educations. The intention is that through such participation educators can recognize the possibility for student learning through simulation. Simultaneously, it is important to acknowledge colleagues’ competence in using other learning methods similar to simulation, e.g., skill-training and role-playing. Many nurse educators are familiar with such methods and may oppose using simulation if they perceive that their approaches are not acknowledged or valued. Student evaluations are used as a strategy to promote simulation in academia and to raise colleagues’ interest. One participant expressed: *“If we ask the students if we should cut out simulation, they immediately answer: No, for all intents and purposes, you must not cut it out”*.

Another way to legitimize SBL in nurse education is by strategically choosing collaborators. Potential collaborators are colleagues from nurse practice or from academia who are familiar with simulation and who support the use of simulation in nursing education, such as preceptors with simulation experience. By integrating simulation in students’ clinical practice, the students’ preceptors become involved, which, in turn, may spark interest and give them ownership to SBL. Through collaborating closely with colleagues in nursing education who are familiar with simulation, and by taking part in formal facilitator education, nurse educators can maintain their engagement, despite experiencing challenges and resistance from leaders or colleagues. Highlighting that the experience as a facilitator can have transmission value to teaching and supervision in general is also a strategy to increase legitimacy amongst colleagues.

Simulation is, however, described as a resource demanding learning activity. Consequently, educators must fight for resources such as time, room, people, and technical support to implement and develop simulation in nurse education. Arguing for resources, particularly with colleagues and leaders who are sceptical of SBL, is important. In addition to fighting for the resources they need to be able to conduct simulation according to good standards, educators also constantly consider how they can optimally use available resources. Using students as peer facilitators or technical assistants (e.g., high-tech equipment or advanced manikins) are ways of responding to resource challenges. Even if this is done under supervision, the strategy reduces teaching staff, and thus saves resources. Additionally, there are economic benefits since student assistants’ wages (in general) are lower than that of educators. Sometimes, the educators have to reduce how many themes and learning outcomes are included in the SBL sessions. If there are other programs where simulation is defined as a learning activity, one strategy is to join forces and offer simulation for mixed groups of students. Also, increasing the number of students in each simulation group to reduce the number of educators is used as a strategy.

The more accepted simulation is in the organisation and the more integrated it is in the curriculum, the more stable  the simulation activities are. Thus, nurse educators do not have to fight for recourses.

##### Self-development

Educators’ self-development is a significant factor contributing to maximizing students’ learning space. The overall strategies are staying up-to-date and seeking continued improvement as facilitators and professional nurses.

Educators need to feel confident in the role as facilitator and with simulation as a learning method. It is therefore important to have systems for mentoring educators, particularly those new to the method. One strategy in this respect is to seek feedback from more experienced peers. As one participant said: *“Then it is not enough to just go through a course and be set to do it alone, and never get feedback on what you are doing […] I have experienced it myself, that when the two of us have been in simulation situations, we can use each other, learn from each other, and get feedback”*.

Peer feedback and collaboration are also important self-developmental strategies for experienced educators. The role as facilitator is challenging and engages the educator at a personal level, as one educator expressed: *“Facilitation is a challenging task. It is also about me as an individual”*. Feedback and collaboration with peers can thus help prevent demotivation and burnout. Developing simulation sequences and conducting simulation together with peers is one way to collaborate. This is, however, resource demanding. Another strategy is to trial the simulation with colleagues before unrolling it to the students. By using this strategy, educators get feedback as facilitators and reassurance that the topics students are to simulate are relevant. Both feedback from equal- or more experienced peers and from less- or unexperienced peers are considered valuable. One way of facilitating this is to invite less experienced colleagues to participate as observers in simulation led by experienced educators, followed by peer discussion.

An important strategy for self-development is attending formal facilitator education. For novice facilitators, it is particularly important to learn how to conduct simulation according to guidelines. Moreover, educators see their first formal facilitator education as a turning point, contributing to confidence in performing simulation with students. For some educators, their first formal facilitator education inspired them to further pursue other relevant conferences and simulation courses, some even at master’s level. All of these strategies facilitate self-development. Educators’ self-development is illustrated by this quote; “*In the beginning, you are occupied by the guidelines and tools. Now, I am flexible, can improvise, and thus handle the situations that must come…*”.

Furthermore, facilitators find it important to be updated professionally, particularly on the topics and cases students are to simulate. Hence, they spend time, sometimes beyond work regulations for self-development as nurse. Participating in courses and conferences with national and international colleagues gives educators a network and access to a simulation community. By engaging in this community, including through research, their self-development continues. This may spark interest and ideas for local research projects. Formal education, experience, and SBL community membership are all important factors to develop an identity and capability as a simulation facilitator.

The more experienced the facilitator becomes, the more capable they are to prepare students and tailor simulation in different scenarios.

##### Preparing students

Many students find simulation anxiety-provoking. Therefore, establishing predictability is important. Facilitators use several strategies to amend this, based on educators’ perceptions of the specific situation and students' involved. Informing students about the learning objectives and the topic prior to simulation is an important strategy and enables students to prepare. Also, educators help students to familiarize themselves with simulation as a learning method in advance, e.g., by explaining and demonstrating the three phases of SBL. An important part of establishing predictability and reducing anxiety is to underline that what happens in simulation is kept confidential. Preparation for simulation is especially important for 1^st^ year students since they are unfamiliar with simulation as a learning method. Facilitators highlight that simulation is a learning method that students need to be introduced to and that educators cannot expect new students to be familiar with the method.

Students become prepared for the simulation sequence by repeating the learning objectives, agreeing on rules, and familiarizing students with the equipment. This is done by strategies such as allowing students to touch and sometimes use some of the equipment and by verbally repeating both expected learning outcomes and rules. Preparing student is particularly important for educators employed in specialised simulation- or learning centres, who usually meet the students for the first time when they come for simulation sessions.

Strategies to increase familiarity and establish predictability are exemplified by one participant saying; *“The reason why... when things go well is that the students […] have been well informed about what they are going to do, and that simulation is not a happening that just “pops up”. We have been in the class and talked about simulation as a learning activity, introduced the case and learning objectives. It’s a good start, in a way. I used to say that simulation is about three phases, and one is the preparation phase. When they come to the simulation, this information is repeated. We let them get to know the equipment, repeat the intended learning outcomes, repeat the expectations, setting the stage. This means that we reduce the stress and that they can carry out the simulation”*.

Highlighting the possibilities for learning and repeating this information when students enter the simulation arena are used as strategies to promote the none-evaluative nature of simulation. For instance, facilitators make it clear for the students that errors in simulation do not affect any evaluations or assessments in other contexts. Mixing of roles where the educator in some situations evaluates students, for example in clinical placements, and in other situations facilitate simulation, can represent a problem for both student and educator. By being aware of students’ vulnerability in simulation in general, and by acknowledging the potential problems of multiple roles, this challenge can be overcome.

##### Tailoring simulation

Helping students experience mastery and promote engagement are key goals in simulation. Strategies to reach this goal are identified under the main category *tailoring simulation*.

To establish authentic learning environments, facilitators try to create simulations that are as realistic as possible. For example, if the simulated case is from an orthopaedical ward, the simulation takes place in a room with hospital beds and equipment relevant in such a ward. Finding simulated patients reflecting the age and sex of the patient case is another strategy to establish authenticity. The less experienced students are, the more authentic the environment should be. This is defined as *high fidelity*.

When the facilitator is aware of students’ emotional state before the simulation sequence takes place, it is easier to organize simulation according to their potential emotional challenges. As an example, anxious students are given a less active role (as assistant or observer) in the beginning and a more active role later in the simulation.

Reflection is a significant part of simulation and essential for learning. Therefore, stimulating and encouraging students to be active during the whole simulation and promoting reflection, particularly during the debrief part, are both considered important. Different strategies are used to promote such reflection. One is giving specific tasks to students in the observer role and asking them to use their observations in the debrief. Observing how the team or the active student assessed the simulated patient’s respiration is an example of such a task. Using “rounds” were all students get the opportunity to talk is another way of involving all students in reflection. Furthermore, facilitators sometimes choose not to interfere if there is an ongoing reflective dialogue among the students. Yet, at the same time, they are being attentive and supportive by using non-verbal communication techniques.

Additionally, highlighting decisive learning episodes is considered important when facilitating reflection. During the simulation sequence, the facilitator uses different strategies to remember what to highlight during the debrief. Taking notes and using different marks, e.g., question marks and exclamation marks, are examples of ways to highlight situations relevant for reflection. Before the debrief, these marks are used to decide what situations ought to be revisited. When prioritizing, the facilitator needs to find a good balance between positive and more adverse or negative situations. This balance is important so that each student is able to walk away from simulation with both mastery experiences and areas for improvement. To make sure students do not leave simulations with a feeling of defeat, facilitators provide students with the opportunity to simulate twice and thus correct mistakes.

All these strategies require the facilitator to be attentive and on the spot. They focus their attention toward two factors: One is the scenario and the equipment, and the other is the communication and interactions within the student group and between student and educator. As one participant expressed: *“I think it’s about having focus, that you’re very focused. You can’t relax, even if the others [students] are training. You’re just as much involved. Because you are the one who must have the overview. You are the one who must pull the strings. You are the one who must put things together. It requires that you are constantly focused on what is happening. […] You know that everyone’s eyes are on you. If [the simulation-sequence] should be positive, it should give them [the students] something professional and social. They should go home and have had a good experience. And then it is certainly necessary to use yourself”*.

By being on the spot, it is possible to adjust simulation according to the defined learning outcomes and to students’ experience. This requires improvisation and the ability to balance support with challenges. By preparing for and tailoring simulation, a safe environment can be established – one that opens up for students’ learning.

## Discussion

The aim of this study was to explore nurse educators’ experiences in facilitating SBL and to develop a grounded theory based on the findings. To our knowledge, this has not been done before in the context of nursing education.

We found that participants’ main concern was to *maximize students’ learning space* in simulation and that the four main categories of legitimizing simulation, self-development as facilitator, preparing students, and tailoring simulations explain how nurse educators strive to maximize the learning space. These categories are summarized in the core category *endeavouring interplay*, which is a useful theoretical framework for SBL.

The four main categories are interrelated. In the following, we present the theory of endeavouring interplay through descriptions of how these categories work together and how facilitators work to optimize students’ learning. Two of the main categories, preparing students and tailoring simulation, focusing on the students, are what nurse educators see as most important in SBL. They are therefore defined as being in the foreground of the theory. The significance of students’ preparation and educators tailoring during simulation is also emphasized in previous research: For instance, studies by Norman [5] and Lee [6] highlight how establishing predictability help students prepare. The importance of establishing a psychologically safe and non-evaluative environment in simulation is emphasized in studies by Kang and Min [8] and Haddeland et al. [20]. Furthermore, how facilitators tailor simulations by being on the spot and adjusting simulation according to participants and context, are the focus of Dieckmann et al. [26] and Madsgaard et al. [25]. Madsgaard et al., focusing on students’ emotions during simulations, found that nurse educators “continuously assess students’ emotional responses” and balanced “levels of difficulty, emotional arousal and psychological safety” ([25], p1). Dieckmann et al. [26] conclude that the interplay among those involved in simulation is one success in simulation. Additionally, the quote “thinking on your feet” put forward by Krogh [19] illustrates the same flexibility we found and describe as “being on the spot”. Moreover, the importance of realism and fidelity are emphasized in several former studies (see Lavoie et al. [29] for an overview).

To summarize: The importance of the two categories defined as the foreground are well documented in former studies and are in line with findings in the theory of endeavouring interplay. The two remaining main categories in the theory, legitimization and self-development, are on the other hand less highlighted in previous studies. We will therefore, in the following, focus more on these two categories. They are significant prerequisites for maximizing students' learning space and thus defined as being in the background of the theory. Legitimization is directed toward the organisation in which simulation is performed and involves several persons and conditions, for example leaders, colleagues, and the history and culture of the organization. Legitimizing is found to be less controllable, quite challenging to work with, and prone to change in order to maximize student learning space. Therefore, to make changes, the nurse educators are striving and using several strategies to increase legitimizing in the organisation.

The other background category, self-development, is more controllable for the nurse educators. Since the focus is on themselves and their own development, these activities are less dependent on contextual issues compared to legitimization. Hence, this category has a more positive nuance as it contributes to competence and mastery and plays an important role in nurse educators’ motivation. A few former studies were found that emphasised facilitators self-development: One study, focusing on manikin-based simulation, underscored the importance of educators being prepared “in terms of the medical content matter in the course, as well as the use of simulation as an educational method” ([30], p715). Interestingly, former studies focusing on legitimizing of simulation in nurse education were not found. There are, however, studies from other educational contexts describing barriers to the implementation of new learning methods. One example is a review from 2021 focusing on Problem-based learning in engineering education, that found implementation challenges at both individual, organisational and cultural level [31].

Our theory describes a reciprocal influence between the foreground and background. Consequently, changes in the background impact the foreground. Low or reduced legitimization of simulation in the organization is likely to reduce nurse educators’ possibilities to tailor simulation and thus minimizes students’ learning space. We found, however, that nurse educators used strategies to buffer such situations. Furthermore, lack of self-development as facilitators can hinder the flexibility nurse educators need to optimize and tailor simulation. This is illustrated by participants describing how they were occupied by performing simulation according to guidelines in the beginning of their career. In such a situation, they may have a higher focus on themselves compared to more experienced educators.

**Fig. 1:**
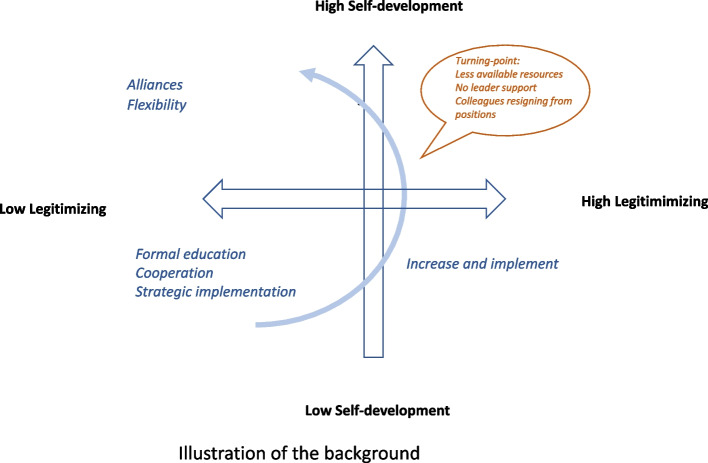
An example of how the background categories, Self-development and Legitimizing, can develop over time

Legitimization and self-development also impact each other: Experienced educators described their pathway from novice to expert facilitator while navigating appropriate strategies for legitimizing SBL. This is illustrated in Fig. 1, where the horizontal axis represents legitimization, and the vertical axis represents self-development (see Fig. 1). The two axes are on a scale from low to high, illustrating their varying presence. Nurse educators’ self-development as facilitators are likely to go from low to high, depending on the strategies affiliated to this category. Legitimization can however take a different path depending on different organisational conditions. As illustrated by the curved arrow in the figure, available resources, leadership support, and curriculum changes may be alerted, thus reducing opportunities for self-development.

Depending on where in this model nurse educators see themselves, they use different strategies. Some strategies are thus more clearly expressed than others in a different zone of the matrix. Fighting for resources and their efforts to develop as facilitators, are examples of strategies applied in the low legitimization and low self-development zone. To participate in research and supervise peers are approaches found in the opposite zone, where legitimization and self-development is high. The pathways are seldom straightforward and were found to shift as organisational climate and opportunities changed, such as changes in staff, reduction of available resources, and weakened leadership support.

A fundamental question arising from our findings, is why nurse educators put so much effort into maximizing the learning opportunities for students in simulation. Here, we need to consider their view on simulation as a learning method that provides closeness to practice and where students can make mistakes without harming real patients. Most SBL facilitators are trained nurse clinicians, which affects their strong sense of obligation to secure quality in patient care and patient safety. This motivation is incorporated into their determination to help students learn.

### Limitations

There are some limitations present in the study. The majority of participants were experienced nurses and SBL facilitators, some were also leaders of learning- or simulation centres. However, they all started as novices and described their development toward experienced facilitators. Another limitation is that one of the researchers has long simulation experience and a possible preconception about the benefit of simulation as a learning method. This is counteracted as the two other has no such experiences. Finally, self-report is seen by some as being less reliable than observation and measurement, but it is also the most effective research approach for areas about which there is very limited knowledge.

## Conclusion

The theory of *Endeavouring interplay* illustrates the complexity educators are encountering when aiming to optimize simulation as a learning space for nurse students. This complexity of being a facilitator is underlined in the Standards of Best Practice that states “A proficient facilitator is required to manage the complexity of all aspects of simulation” ([10], p23). The strategies used are adapted to the organisational climate, available resources and context, and include striving to legitimize simulation, pursue self-development in the role as facilitator, help students prepare for SBL, and tailor the simulation to both contextual factors and individual student needs.

## Implications for practice, education, theory, and research

The theory of *Endeavouring interplay* demonstrates a framework that can apply to both clinical practice and education, both for students and working nurses. While the complexity of being a facilitator in nurse education is clearly underlined in this theory, the strategies presented by participants can be replicated in both areas, and the theory provides a sound framework for development of knowledge and skills. The theory shows how the four categories affect each other and thus illustrate the complexity of nurse educators’ experience with SBL. No previous studies were found that combine how educators work to maximize student learning space by preparing students, tailoring simulation, legitimizing simulation, and develop as facilitators; therefore, more research is needed in this area, including testing the theory of *Endeavouring interplay* in other sites and learning situations.

### Supplementary Information

**Additional file 1. **Interview guide.

## Data Availability

The data that support the findings of this study are available from Kari Røykenes but restrictions apply to the availability of these data, which were used under license for the current study, and so are not publicly available. Data are however available from the first author upon reasonable request and with permission of the individual interviewee.
