# Supplementary material for: Endeavouring interplay: a grounded theory study of how nurse educators’ work with simulation-based learning
Source: BMC Nurs. 2023 Oct 11;22:377. doi: 10.1186/s12912-023-01546-9 (PMC10568800; doi:10.1186/s12912-023-01546-9)
Supplement: Supplementary file 1 — Additional file 1. Interview guide. [file 12912_2023_1546_MOESM1_ESM.docx]

**Endeavouring interplay. How nurse educators’ work with simulation-based learning. A Grounded theory study.**

**Interview guide:**

Please tell us about your experiences with using simulation (from a facilitator perspective).

Can you tell us about when you started using simulation-based learning?

What makes you still use this method?

What do you see as the advantages of this way of acquiring knowledge?

Are there any simulations situations that have had a strong effect on you?

Possible probing question: If so, could you please tell us about one or more of them?

What are your thoughts on the role as facilitator?

What do you think about the different role you might have in a simulation sequence?

What is necessary for a simulation to work well?

What tips and advice would you give to a teacher who is new to simulation?

**Added questions (theoretical sampling):**

Can you give examples of sequences of simulation that worked well and why you think they did?

Possible probing question: Can you describe them and what you think made them successful?

Can you give examples of situations where simulation did not work as intended?

Possible probing question: What do you think might have been the reason for that?

How do you work to advocate and legitimize SBL at your university?

How do you work with your competence as facilitator?

How do you assess when you ought to be supportive and when you can trigger students in simulation?

What motivates you to continue using simulation as a learning method?
